# Treatment of scleredema adultorum of Buschke with intravenous immunoglobulin and mycophenolate mofetil in a 14-year-old girl: a case report

**DOI:** 10.1186/s13256-024-04427-0

**Published:** 2024-02-14

**Authors:** Mehran Pournazari, Dena Mohamadzadeh, Shirin Assar, Mazaher Ramezani

**Affiliations:** 1 Clinical Research Development Center, Imam Reza Hospital, Kermanshah University of Medical Sciences, Kermanshah, Iran; 2 Student Research Committee, Kermanshah University of Medical Sciences, Kermanshah, Iran; 3 Molecular Pathology Research Center, Imam Reza Hospital, Kermanshah University of Medical Sciences, Kermanshah, Iran

**Keywords:** Scleredema adultorum of Buschke, Skin thickening, Intravenous immunoglobulin, Mycophenolate mofetil

## Abstract

**Background:**

Scleredema adultorum of Buschke is a rare disease characterized by firm and non-pitting edema of the skin. The condition is rare with unknown etiology. Diagnosis is made on the basis of clinical findings and skin biopsy.

**Case presentation:**

Here, we describe a 14-year-old Iranian girl presenting with non-pitting edema and woody thickening of the skin that progressed within a month. She was evaluated for possible underlying malignancy or connective tissue disorders, which were excluded by multiple laboratory workups. She underwent a skin biopsy which confirmed the diagnosis of scleredema, and she was successfully treated with intravenous immunoglobulin and mycophenolate mofetil.

**Conclusion:**

While scleredema adultorum of Buschke is a rare disease with no definite treatment, our effort through this report was to highlight the possible benefits of treatment by intravenous immunoglobulin and mycophenolate mofetil.

## Background

Scleredema adultorum of Buschke is a rare disease believed to be in the spectrum of scleroderma-like disorders. The etiology of the disease is not well understood. Curizo first described this condition in 1752. But the term “scleredema adultorum of Buschke” is attributed to Abraham Buschke, who defined the disease in 1902. The condition is characterized by non-pitting edema and woody induration of the skin. The common sites of skin involvement are the face, neck, trunk, and limbs, but hands and feet are typically spared. The diagnosis is made on the basis of clinical manifestations and histological findings. No laboratory test has been suggested yet. Skin biopsy shows edematous spaces between thickened collagen bundles. The epidermis is intact. While various therapeutic methods have been tried, there is no definite treatment yet [1–3].

## Case presentation

A 14-year-old Iranian female presented to the outpatient clinic of rheumatology at Kermanshah University of Medical Sciences in 2017. She complained of thickening, itching, and swelling of her skin, which progressed within 1 month and involved the trunk and lower and upper limbs with sparing of face, hands, and feet. She had no history of diabetes mellitus or febrile sickness before the initiation of skin thickness. She denied Raynaud’s phenomenon, dysphagia, gastroesophageal reflux, dyspnea, and cough. Her past medical history and familial history were negative for autoimmune or connective tissue disorders.

Before this meeting, she had been evaluated in hematology and dermatology clinics for possible underlying malignancy and hypereosinophilic syndrome due to serum eosinophilia. At that time laboratory findings included hemoglobin of 14.4 mg/dl, white blood cell count (WBC) of 8.6 × 10^3^/mm^3^ (differential count: neutrophils 59.8%, lymphocyte 29.5%, eosinophil 10.7%), platelet count of 264 × 10^3^/mm^3^, and normal serum protein electrophoresis. Thyroid-stimulating hormone (TSH) was within normal limits. Human immunodeficiency virus (HIV) antibody, hepatitis C virus (HCV) antibody, and hepatitis B surface (HBS) antigen were negative. Abdominopelvic sonography was unremarkable. Tumor markers (CEA, CA-125, CA 19-9, CA 15-3, AFP) were negative. Bone marrow aspiration and biopsy were normal. A polymerase chain reaction (PCR) experiment for *JAK-2* mutation was carried out, and the result was negative. Genetic evaluation for PDGFRA, PDGFRB, and FGFR1 rearrangements was normal; therefore, hypereosinophilic syndrome was excluded.

**Fig. 1:**
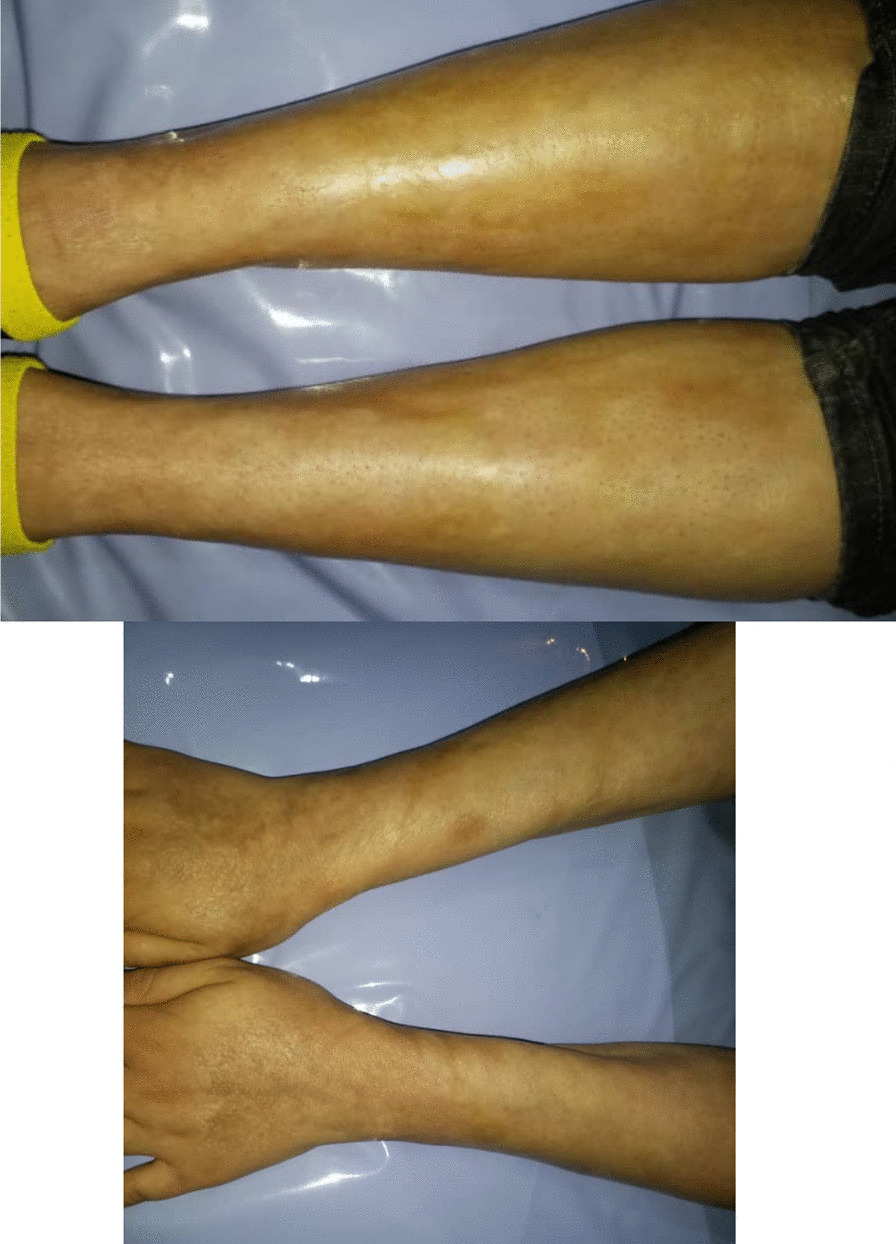
Showing the skin thickening and swelling of the legs and forearms of the patient and areas of hyperpigmentation

Additional workup was done in our rheumatology clinic. Physical examination was unremarkable except for generalized skin thickening, swelling, and hyperpigmentation with sparing of hands and feet (Fig. 1). Echocardiography was normal (ejection fraction = 60% and pulmonary artery pressure = 15 mmHg). A capillaroscopy was performed, and the findings were non-significant. Immunological tests were done. Antinuclear antibody (ANA), anti-double-stranded deoxyribonucleic acid (anti-ds DNA) antibody, anti-SCL-70 antibody, anti-centromer antibody, perinuclear and cytoplasmic anti-neutrophil cytoplasmic antibody (P & C-ANCA), anti-Ro, anti-La, angiotensin-converting enzyme (ACE), C3, C4, CH50, anti-cyclic citrullinated peptide (anti-CCP), and rheumatoid factor (RF) were negative.

**Fig. 2:**
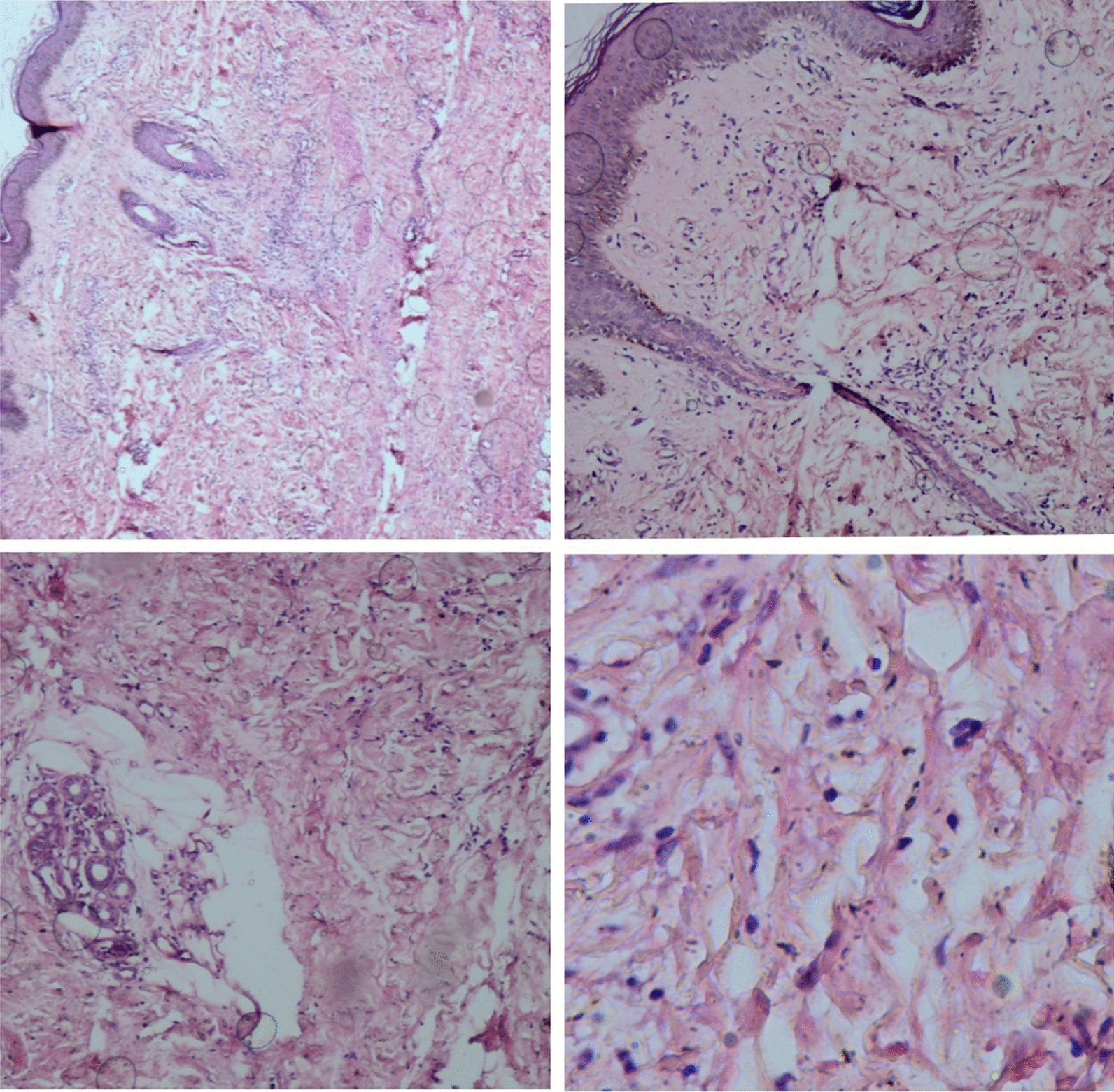
Skin biopsy specimen from right forearm (hematoxylin–eosin stain, × 40, 100, 400): Figures show excessive and thickened collagen bundles separated by edematous spaces in the dermis. The epidermis and eccrine ducts are intact

A skin biopsy was taken from the right forearm, which showed a thickened dermis with excessive collagen fibers separated by wide edematous spaces. Epidermis and eccrine ducts were unaffected (Fig. 2). Diagnosis of scleredema adultorum of Buschke was confirmed, and the patient was started on prednisolone 1 mg/kg daily with gradual tapering to 10 mg/day and methotrexate 15 mg/weekly. No significant improvement was seen after 3 months. Therefore, methotrexate (MTX) was replaced by mycophenolate mofetil 2000 mg/daily. And 2 g/kg of intravenous immunoglobulin (IG) was administered in four divided doses. Gradual improvement was reported by the patient, and resolution of the skin thickness and induration was observed by expert rheumatologist. After 5  years, she is still under follow-up in the rheumatology clinic and maintains on prednisolone 2.5 mg daily and methotrexate 10 mg weekly. Her skin thickness has been improved significantly.

## Discussion and conclusion

Scleredema adultorum of Buschke manifests via firm, non-pitting edema that does not involve the distal limbs. The disease is classified into three types. Type 1 has been known as the classic type, which was first described by Buschke. This type is typically preceded by a febrile illness such as influenza and resolves completely within months to 2 years [1]. In contrast, type 2 is not preceded by a febrile sickness. This type progresses slowly and is associated with paraproteinemias including multiple myeloma [4, 5]. Type 3 is associated with diabetes mellitus and, similar to type 2, is slowly progressive and has a non-resolving course [6]. In our patient, skin thickness developed rapidly within 1 month and was not completely resolved after 5 years. Our patient did not have diabetes mellitus and workup for malignancy, including serum protein electrophoresis, returned negative. She denied any febrile disease before the onset of symptoms. Therefore, our patient did not fit in any of the mentioned types. Other diseases known to be associated with scleredema of Buschke include primary hyperparathyroidism, rheumatoid arthritis, Sjogren’s syndrome, ankylosing spondylitis, dermatomyositis, and Waldenstrom macroglobulinemia. No sign or symptom of these conditions was found in our patient [1–3].

The most important differential diagnosis of scleredema of Buschke includes scleromyxedema and scleroderma. Scleroderma is the thickening of the skin observed in systemic sclerosis, which typically initiates from hands and feet. Distinct features of scleroderma include Raynaud phenomenon, abnormal capillaroscopy, and the presence of specific autoantibodies. Scleromyxedema is also a part of scleroderma-like disorders. It is characterized by skin papules and plaques, which could involve all parts of skin, including hands and feet. Dermal fibrosis is observed in skin biopsy separated by mucin deposits [1, 2, 7].

The role of skin biopsy is to confirm the diagnosis and exclude other differential diagnoses. Histological findings include thickened collagen fibers, separated by mucin-filled spaces. Infiltration of mononuclear lymphocytes is observed around capillaries. The subcutaneous fat is invaded by thickened collagen bundles. The epidermis is normal [1, 2].

While various therapeutic methods have been tried, there is no definite treatment yet. Treating the underlying disease is first-line therapy: antibiotic therapy for bacterial infections preceding scleredema type 1, treatment of the underlying hematologic disorder in type 2, and management of diabetes in type 3. Phototherapy could be useful. Other treatments include steroids, cyclosporine, cyclophosphamide, and intravenous penicillin [8–11]. Because our patient did not respond to MTX, we decided to use another immunosuppressive agent, and we chose mycophenolate mofetil. Intravenous immunoglobulin (IG) could be beneficial in severe cases, as used in our case. Intravenous IG has been efficient in various inflammatory diseases, but its mechanism of action remains elusive. It might have immunomodulatory and anti-inflammatory effects by suppression of pro-inflammatory cytokines, downregulation of adhesion molecules and chemokine receptors, and neutralization of super-antigens [2, 12–15].

Rongioletti *et al*. studied 44 patients with scleredema. Of these, 30 patients had DM, and 5 had monoclonal gammopathies. Phototherapy was effective in mild cases. Corticosteroids and immunosuppressive therapy were used in severe cases. After a mean period of 32 months, 39 patients were still alive, and of these 9 did not have skin thickness anymore [2].

While scleredema adultorum of Buschke is a chronic and debilitating disorder, and there is no definite treatment. Various therapeutic methods have been tried and could be beneficial. Intravenous immunoglobulin may be a potentially effective treatment in severe and unresponsive treatments to corticosteroids and immunosuppressive drugs.

## Data Availability

Available if requested.

## Abbreviations

IG: Immunoglobulin
ANA: Antinuclear antibody
anti-ds DNA: Anti-double-stranded deoxyribonucleic acid
P & C-ANCA: Perinuclear and cytoplasmic anti-neutrophil cytoplasmic antibody
anti-CCP: Anti-cyclic citrullinated peptide
RF: Rheumatoid factor

## References

[1] Venencie PY, Powell FC, Su WD, Perry HO (1984). Scleredema: a review of thirty-three cases. J Am Acad Dermatol.

[2] Rongioletti F, Kaiser F, Cinotti E, Metze D, Battistella M, Calzavara-Pinton P (2015). A multicentre study of characteristics, comorbidities, course and therapy in 44 patients. J Eur Acad Dermatol Venereol.

[3] Beers WH, Ince A, Moore TL, editors. Scleredema adultorum of Buschke: a case report and review of the literature. Seminars in arthritis and rheumatism; 2006: Elsevier. 10.1016/j.semarthrit.2006.01.004 16765712

[4] Krejci M, Adam Z, Pour L, Michalkova E, Sandecka V, Szturz P (2015). Scleredema associated with multiple myeloma or MGUS: treatment report of four cases. Clin Lymphoma Myeloma Leuk.

[5] Barnes M, Kumar V, Le T-H, Nabeel S, Singh J, Rana V (2020). A case of paraproteinemia-associated scleredema successfully treated with thalidomide. JAAD Case Rep.

[6] Ezejiofor O, Onayemi O, Olasode O, Ikem R, Komolafe A, Ezejiofor I (2015). Scleredema diabeticorum—a case report. J Dermatol Dermatol Surg.

[7] Gabrielli A, Avvedimento EV, Krieg T (2009). Scleroderma. N Engl J Med.

[8] Hager CM, Sobhi HA, Hunzelmann N, Wickenhauser C, Scharenberg R, Krieg T (1998). Bath-PUVA therapy in three patients with scleredema adultorum. J Am Acad Dermatol.

[9] Kurtoğlu S, Yüksel Ş, Gündüz Z, Per H, Narin N, Kontaş O (2004). Use of high-dose intravenous corticosteroid treatment in a child with scleredema. J Emerg Med.

[10] Skrepnik T, Gottesman S, Stea B (2016). Long-term benefit of electron beam radiation therapy in the treatment of scleredema of Buschke. Adv Radiat Oncol.

[11] Mattheou-Vakali G (1996). Cyclosporine in scleredema. J Am Acad Dermatol.

[12] Miguel D, Schliemann S, Elsner P (2018). Treatment of scleroedema adultorum Buschke: a systematic review. Acta Derm Venereol.

[13] Aichelburg MC, Loewe R, Schicher N, Sator PG, Karlhofer FM, Stingl G, Jalili A (2012). Successful treatment of poststreptococcal scleredema adultorum Buschke with intravenous immunoglobulins. Arch Dermatol.

[14] Linares-González L, Ródenas-Herranz T, Espelt-Otero JL, Ruiz-Villaverde R. Buschke sclerederma refractory to conventional treatment: response to UV-A1 phototherapy. 10.1016/j.ad.2019.10.011 33253645

[15] Eastham AB, Femia AN, Velez NF, Smith HP, Vleugels RA (2014). Paraproteinemia-associated scleredema treated successfully with intravenous immunoglobulin. JAMA Dermatol.

